# Statistical machine learning models for prediction of China’s maritime emergency patients in dynamic: ARIMA model, SARIMA model, and dynamic Bayesian network model

**DOI:** 10.3389/fpubh.2024.1401161

**Published:** 2024-06-27

**Authors:** Pengyu Yang, Pengfei Cheng, Na Zhang, Ding Luo, Baichao Xu, Hua Zhang

**Affiliations:** ^1^Department of Nursing, West China Hospital, Sichuan University, Chengdu, China; ^2^Department of Nursing, The Second Affiliated Hospital of Zhejiang University School of Medicine, Hangzhou, China; ^3^International Nursing School, Hainan Medical University, Haikou, China; ^4^Department of Physical Education, Hainan Medical University, Haikou, China; ^5^Hainan Provincial Key Laboratory of Sports and Health Promotion, Hainan Medical University, Haikou, China

**Keywords:** medical assistance at sea, prediction, ARIMA, SARIMA, emergency medical service, dynamic Bayesian network

## Abstract

**Introduction:**

Rescuing individuals at sea is a pressing global public health issue, garnering substantial attention from emergency medicine researchers with a focus on improving prevention and control strategies. This study aims to develop a Dynamic Bayesian Networks (DBN) model utilizing maritime emergency incident data and compare its forecasting accuracy to Auto-regressive Integrated Moving Average (ARIMA) and Seasonal Auto-regressive Integrated Moving Average (SARIMA) models.

**Methods:**

In this research, we analyzed the count of cases managed by five hospitals in Hainan Province from January 2016 to December 2020 in the context of maritime emergency care. We employed diverse approaches to construct and calibrate ARIMA, SARIMA, and DBN models. These models were subsequently utilized to forecast the number of emergency responders from January 2021 to December 2021. The study indicated that the ARIMA, SARIMA, and DBN models effectively modeled and forecasted Maritime Emergency Medical Service (EMS) patient data, accounting for seasonal variations. The predictive accuracy was evaluated using Mean Absolute Error (MAE), Root Mean Squared Error (RMSE), and Coefficient of Determination (*R*^2^) as performance metrics.

**Results:**

In this study, the ARIMA, SARIMA, and DBN models reported RMSE of 5.75, 4.43, and 5.45; MAE of 4.13, 2.81, and 3.85; and *R*^2^ values of 0.21, 0.54, and 0.44, respectively. MAE and RMSE assess the level of difference between the actual and predicted values. A smaller value indicates a more accurate model prediction. *R*^2^ can compare the performance of models across different aspects, with a range of values from 0 to 1. A value closer to 1 signifies better model quality. As errors increase, *R*^2^ moves further from the maximum value. The SARIMA model outperformed the others, demonstrating the lowest RMSE and MAE, alongside the highest *R*^2^, during both modeling and forecasting. Analysis of predicted values and fitting plots reveals that, in most instances, SARIMA’s predictions closely align with the actual number of rescues. Thus, SARIMA is superior in both fitting and forecasting, followed by the DBN model, with ARIMA showing the least accurate predictions.

**Discussion:**

While the DBN model adeptly captures variable correlations, the SARIMA model excels in forecasting maritime emergency cases. By comparing these models, we glean valuable insights into maritime emergency trends, facilitating the development of effective prevention and control strategies.

## 1 Introduction

Working at sea is one of the world’s most perilous occupations, characterized by a markedly high accident rate (1). Surveys reveal that there are 103 injuries per 1,000 full-time fishermen (2). In Australia, the annual fatality rate at sea is three times that of agriculture and four times that of the road freight sector (1). Similarly, in the United Kingdom, the fatal accident rate at sea from 2003 to 2012 was nearly five times higher than in the construction sector (3), highlighting the significant risk and mortality associated with maritime accidents. Delivering emergency care at sea presents considerable challenges exacerbated by factors such as geographic isolation, limited medical personnel, remoteness, and the scarcity of medical facilities aboard ships (4). These conditions render medical emergencies at sea among the most arduous prehospital situations for healthcare professionals and researchers (5). Furthermore, low- and middle-income countries encounter difficulties in providing maritime and aquatic first aid, contributing to a major global public health concern. According to Dykes et al. (6), approximately 19% of all marine search and rescue missions require medical evacuation at sea, significantly impacting patient outcomes due to delays in rescue operations and the absence of immediate responders.

To tackle the aforementioned challenges, the United States has initiated several telemedical maritime assistance service centers and extended telemedicine globally since 2003 to alleviate the shortage of medical personnel at sea (7). Scholars like Adrian P (8). have scrutinized sea and land ambulance response times in the Philippines, devising care strategies and spatial pathways to expedite detection, optimize human resource allocation, and reduce rescue durations. However, dynamically adjusting medical rescue personnel at sea remains an unresolved issue. Zhang et al. (9) have proposed dynamically allocating emergency resources; yet existing models struggle with uncertain data and lack the capability to adapt human resources promptly. The demand for Emergency Medical Services (EMS) at sea fluctuates on a monthly basis, particularly during periods of heightened migrant, refugee, and asylum seeker rescues, or mass casualties from disasters (10–13). Leveraging historical data to forecast future requirements aids in improved scheduling and staffing, thereby enhancing emergency supply reserves for high-risk days. Historical data has proven instrumental in predicting demand and understanding variability, facilitating more effective pre-hospital emergency care planning (14). Accurate prediction of human resource needs enables hospitals to circumvent the expenses associated with hiring temporary staff or implementing flexible schedules, thus reducing response times and resource wastage. The development of a dynamic scheduling system that aligns with patient needs, catering to both full-time and part-time staff, holds immense promise in addressing these challenges. Hence, the creation of a precise model for predicting emergency patient numbers at sea is paramount for optimizing medical manpower allocation and shaping preventive measures and policies.

Recent research has increasingly embraced advanced technologies such as deep learning, neural networks, and big data for forecasting patient volumes. Key models in this domain include the Auto-regressive Integrated Moving Average (ARIMA), Seasonal Auto-regressive Integrated Moving Average (SARIMA), and Dynamic Bayesian Networks (DBN). ARIMA, renowned for its efficiency in capturing linear trends in time series data with minimal computational burden, is often employed to explore variable relationships or serve as a benchmark in testing hybrid models, albeit with mixed outcomes (15). For instance, Li et al. (16) in China utilized ARIMA to assess the impact of the Corona Virus Disease 2019 (COVID-19) on gonorrhea trends, while Eyles et al. (17) in the United Kingdom (UK) employed it to generate precise short-term forecasts for patient admissions and bed occupancy, thereby improving predictions for medical specialties and lengths of stay. And ARIMA has proven capable of processing and predicting complex but stable time series data. It can also effectively manage short-term mutations and trends, making it suitable for rapid prediction of emergency events (18). Despite demonstrating high accuracy, ARIMA may struggle with seasonal fluctuations, potentially leading to prediction inaccuracies. SARIMA, on the other hand, excels in analyzing time series periodicity, trends, and disturbances, making it a staple choice for infectious disease forecasting (19–21). Almeida et al. (22) utilized SARIMA to study pediatric emergency department visits, while Zhang et al. (23) applied it to predict hospital blood demand, facilitating resource allocation. Moreover, the maritime environment has significant seasonal characteristics, such as monsoons and tides, so the SARIMA model can better capture and predict these cyclical changes. However, SARIMA’s limitations in capturing dynamic inter-variable changes can impede prediction accuracy. In contrast, the DBN model, a graphical representation of variable correlations and temporal changes (24), finds extensive application in infectious disease (25–27) and diabetes research (28) for pathway identification and risk assessment. Emergencies at sea often involve multiple uncertain factors, such as weather changes, wave conditions, ship status, etc. The DBN model can effectively process and integrate this information for real-time dynamic prediction. Despite its utility, DBN typically requires at least 3 years of historical data (25), compared to ARIMA and SARIMA models, which necessitate a minimum of 50 time points (15). Consequently, while DBN models demand less data, they may be susceptible to specific errors. Furthermore, the comparative effectiveness of these models in forecasting emergency patient volumes and their sensitivity to dynamic predictions remain relatively unexplored.

In this study, we delved into the stability and predictive efficacy of three models to analyze the number of maritime emergency patients. By comparing the fitting and predictive abilities of these models, our goal is to provide an early warning system, facilitating effective prevention and control strategies for maritime emergencies. This includes ensuring timely allocation of manpower and medical resources in anticipation of significant maritime incidents along China’s coast. First, this study utilized medical records from the South China Sea region of Hainan Province spanning from 2016 to 2020 to develop ARIMA, SARIMA, and DBN models. These models were evaluated using 2021 data to improve the analysis and prediction of illness trends among offshore emergency patients. The rest of this document is organized as follows: Section 2 outlines the data collection locations, time frames, ethical considerations, inclusion and exclusion criteria, data entry methods, statistical models, their fundamental principles, analysis tools, evaluation criteria, and the overall modeling process. Section 3 elaborates on the modeling procedures of the three models, the selection of parameters, the comparison of their predictive performance, and the identification of the best model. In addition, Section 4 elaborates on why SARIMA excels in predicting the number of first aid workers at sea, the importance of forecasting marine emergencies, the strengths and limitations of this study, and recommendations for future policy. Finally, Section 5 presents the conclusions derived from this research.

## 2 Subjects and methods

### 2.1 Subjects

The researcher discovered through initial investigations that the Hainan Maritime Safety Bureau primarily directs sea medical rescues in the South China Sea to five key medical facilities: Haikou City’s first aid center handles the Haikou region, while Sanya City, Dongfang City, Wenchang City, and the Yangpu Economic Development Zone each have a dedicated hospital for other areas. Consequently, maritime emergency patients treated at these five Hainan Province hospitals between January 2016 and December 2021 were chosen for this study. Previous research (15, 25) indicates that constructing ARIMA, SARIMA, and DBN models requires a minimum of 50 time points and 3 years of historical data, respectively. Accordingly, this study utilized EMS patient data from January 2016 to December 2020 for model development, and data from January to December 2021 served as an internal validation set to assess the models’ predictive accuracy. The Hainan Medical University Ethics Committee approved this study (NO.: HYLL-2022-018), adhering to the Declaration of Helsinki’s guidelines. Informed consent was obtained from all participants.

### 2.2 Data collection

#### 2.2.1 Inclusion criteria

In this study, we examine various cases including: ① 120 instances of emergency vehicles arriving at the dock, port, harbor first aid station, etc.; ② communication with units such as the Maritime Bureau, Coastal Radio Division, port medical aid station, etc.; ③ the nature of calls involving ships affected by natural disasters like typhoons, as well as other onboard emergencies such as poisoning, requiring immediate medical attention for patients, sudden illnesses or injuries, and the medical history of patients describing injuries or the onset of illnesses onboard, such as cable strangulation injuries, acute gastric perforation, and drowning due to ship sinking or jumping into the sea.

#### 2.2.2 Exclusion criteria

This study excludes: ① 120 instances where emergency vehicles arrived at the scene but did not encounter the patient; ② medical records containing missing information, incomplete data (missing more than 3 items), errors, and duplicates; ③ patients who independently visited the hospital for prescriptions, examinations, or consultations without recorded statistics are also excluded.

#### 2.2.3 Data entry

From the pre-hospital case management system of the five hospitals mentioned above, data of emergency patients at sea in the South China Sea from 2016 to 2021 were exported. If the hospital did not enter into the pre-hospital case management system in that year, the data was collected by manually flipping through records and using image records (taken by the camera) to gather relevant information. Afterward, a database was set up using Excel software to conduct the study, as shown in Table 1.

**Table 1:** Emergency medical treatment at sea in Hainan region from January 2016 to December 2021.

Month	Jan.	Feb.	Mar.	Apr.	May	Jun.	Jul.	Aug.	Sep.	Oct.	Nov.	Dec.
Year												
2016	18	14	20	19	16	13	10	17	17	17	29	20
2017	20	15	15	17	21	11	6	39	18	21	25	18
2018	16	18	16	18	15	7	6	13	21	21	19	19
2019	20	14	20	19	12	3	6	13	17	24	28	27
2020	31	27	29	19	15	9	13	16	25	23	27	22
2021	22	19	22	21	17	8	8	16	20	24	26	25

### 2.3 Statistical models and description

First, the data were loaded and preprocessed to create trend charts and related factor decomposition charts, and then a time series model was developed to forecast sea emergency patients using three predictive models: ARIMA, SARIMA, and DBN. The prediction framework for the time series model is depicted in Supplementary Figure 1.

#### 2.3.1 ARIMA (*p*, *d*, *q*)

The ARIMA model is commonly represented as ARIMA (p, d, q), where Auto-Regressive (AR) signifies the auto-regressive function, I represents the differencing term, and Moving Average (MA) stands for the moving average function. Here, *p* indicates the count of autoregressive terms, *q* represents the number of moving average terms, and *d* signifies the levels of differencing applied to transform the original dataset into a smoother series. Below are the generalized formulas for the *p*-order AR model [Equation (1)] and the *q*-order MA model [Equation (2)].


(1) $$AR\left(p\right):Y_{t}=\mu +\beta_{1}Y_{t-1}+\beta_{2}Y_{t-2}+\cdots +\beta_{p}Y_{t-p}+\varepsilon_{t}$$

In this sequence, every value can be depicted as a linear combination of its preceding p values. Here, $Y_{t}$ represents any given observation within the sequence, μ stands for the sequence’s average, β denotes the weight, and $\varepsilon_{t}$ signifies the random disturbance.


(2) $$MA\left(q\right):Y_{t}=\mu -\theta_{1}\varepsilon_{t-1}-\theta_{2}\varepsilon_{t-2}\ldots -\theta_{q}\varepsilon_{t-q}+\varepsilon_{t}$$

where each value of the sequence can be represented as a linear combination of the previous q residuals. ε denotes the predicted residuals and θ is the weight.

The ARMA (*p*, *q*) model merges the AR model [Equation (1)] with the MA model [Equation (2)], resulting in a unified model expressed mathematically as Equation (3).


(3) $$\begin{array}{c} Y_{t}=\mu +\beta_{1}Y_{t-1}+\beta_{2}Y_{t-2}+\cdots +\beta_{p}Y_{t-p}-\theta_{1}\varepsilon_{t-1}\\ -\theta_{2}\varepsilon_{t-2}\cdots -\theta_{q}\varepsilon_{t-q}+\varepsilon_{t} \end{array}$$

where each sequence value is depicted as a linear blend of p previous observations and *q* residuals.

The ARIMA (*p*, *d*, *q*) model assumes that the time series data is non-stationary. The integration (I) component involves differencing the data to meet the model’s requirements for smoothness, allowing further steps in the modeling process. After differencing the data d times, each point in the series is modeled as a linear combination of p past observations and q residuals. This process, which ensures the data adheres to the smoothness criterion, precedes the ARMA (*p*, *q*) modeling, represented by the subsequent Equation 4:


(4) $$\begin{array}{c} Y_{t}{\;}^{\prime }=\mu +\beta_{1}Y_{t-1}{\;}^{\prime }+\beta_{2}Y_{t-2}{\;}^{\prime }+\cdots +\beta_{p}Y_{t-p}{\;}^{\prime }\\ -\theta_{1}\varepsilon_{t-1}-\theta_{2}\varepsilon_{t-2}\cdots -\theta_{q}\varepsilon_{t-q}+\varepsilon_{t} \end{array}$$

where $Y_{t}{\;}^{\prime }$ represents the differenced sequence.

#### 2.3.2 SARIMA (*p*, *d*, *q*) (*P*, *D*, *Q*)

Data often exhibit seasonal trends, making a simple ARIMA model inadequate for capturing their correlations. This necessitates employing SARIMA, which further differentiates the data based on the time series’ seasonal cycle, typically denoted as SARIMA (*p*, *d*, *q*) (*P*, *D*, *Q*)_[*s*]_. In this notation, *P* represents the number of seasonal autoregressive terms, *D* the number of seasonal differencing orders, *Q* the number of seasonal moving average terms, and *s* the length of the seasonal cycle. The general SARIMA model is mathematically expressed as follows (Equation 5):


(5) $$\Phi_{Ρ}\left({Β}^{m}\right)\varphi_{\rho }\left(Β\right){\left(1-{Β}^{m}\right)}^{D}{\left(1-Β\right)}^{d}Yt=\Theta_{Q}\left({Β}^{m}\right)\theta_{q}\left(B\right)w_{t}$$

Where Yt represents the non-stationary time-series, $W_{t}$ stands for the Gaussian white noise process, $\varphi \left(B\right)$ denotes the non-seasonal auto-regressive polynomial, and $\theta \left(B\right)$ signifies the non-seasonal moving average polynomial. Additionally, *D* represents the seasonal differencing term, which can adopt the values 1 or 2, among others. However, the value of *D*1 effectively guarantees the data’s stationarity. Furthermore, $\Phi_{P}\left(B^{m}\right)$ symbolizes the seasonal auto-regressive polynomial, while $\Theta_{Q}\left(B^{m}\right)$ represents the seasonal moving average polynomial. In this context, *B* is defined as the backshift operator, described as follows (Equation 6):


(6) $$B^{k}Y_{t}=Y_{t-k}$$

#### 2.3.3 DBN model

The Least Absolute Shrinkage and Selection Operator (LASSO) regression algorithm stands out as a method for variable selection, known for its high model stability. It systematically reduces coefficients by incorporating penalty terms during model estimation, streamlining the model and addressing overfitting and multicollinearity effectively (29). Tibshirani (30) is renowned for pioneering the LASSO approach, enabling simultaneous variable selection and coefficient estimation. The LASSO estimate is identified as the solution to Equation 7.


(7) $$\left(Y-X\beta \right)\beta \min{\;}^{\prime }\left(Y-X\beta \right)+\lambda \overset{k}{\underset{j=1}{\sum }}\left|\beta_{j}\right|,\lambda \geq 0$$

Least Absolute Shrinkage and Selection Operator regression becomes equivalent to ordinary least square (OLS) regression when the tuning parameter λ is set to 0. As the tuning parameter λ grows, it progressively reduces the magnitude of the unknown regression parameter vector β toward 0, leading to some regression parameters being precisely reduced to 0 for sufficiently high values of λ . The predictors linked to these zero-valued regression parameters are deemed inactive and are consequently excluded from the model.

The configuration and parameters of a DBN are typically derived by employing the LASSO algorithm (25). This method operates on the principle that, by introducing an L1 penalty to the least squares minimization function, it is possible to reduce the coefficients of less correlated variables to zero. This process refines the model by eliminating irrelevant variables, leaving only significant variables represented by directed arcs to the response variables. On the other hand, Bayesian Networks (BNs), a type of directed acyclic graph (DAG), depict the relationships between variables using nodes and arcs, with nodes symbolizing random variables and arcs illustrating the interactions among them. Given that complex systems evolve over time, static BN models fall short in capturing these dynamics, leading to the development of DBNs. These models extend BNs by incorporating time, allowing for the analysis of time series data through arcs that connect variables at successive time points. This feature enables DBNs to capture both the interactions among variables and how these relationships evolve over time, offering a comprehensive framework for understanding dynamic systems. The calculation method is shown in Equation 8.


(8) $${Χ}_{t}=u_{t}+A_{1}X_{t-1}+\cdots +A_{p}X_{t-p}+a_{t}$$

Let (*t* = 1, 2, \ldots, T) where ${Χ}_{t}=\left(X_{i\left(t\right)}\right)$ , (*i* = 1, \ldots, k), represents (*k*) as the vector of observations of the number of first aiders at sea at time (*t*). (A) is the matrix of coefficients to be estimated, and $X_{t-p}$ represents the vector of observations of the number of first aiders at sea at lag order (p). $u_{t}$ denotes the constant term, and $a_{t}$ represents the residuals. All arcs in the network are defined between two consecutive time points, with the set of arcs represented by the matrix $A_{c}\left(1\leq c\leq t\right)$ . If an element $a_{ij}\neq 0\;\left(i\neq j\right)$ exists in $A_{c}$ , then the network includes an arc from $X_{i\left(t-c\right)}X_{j\left(t\right)}$ .

### 2.4 Analytical tools and model evaluation

The following tools are employed to evaluate the reliability of time series analysis: Auto-Correlation Function (ACF), Partial Auto-Correlation Function (PACF), Augmented Dickey-Fuller Test (ADF test), Akaike’s Information Criterion (AIC), and Ljung-Box Test (LB test). These tools help identify the relationships between observations in a time series. ACF measures the correlation between the time series data and its previous values, whereas PACF identifies the correlation of the time series with its lagged values by specific time intervals. The ADF test assesses the smoothness of the time series, with the trend term parameter indicating the significance of the trend; a non-smooth series requires differencing before applying the ADF test. AIC serves as a penalized likelihood criterion, with a lower AIC value suggesting a more plausible model. The LB test checks if the series is purely random, using the *p* value to determine the presence of white noise.

The metrics employed in this study included Relative Error (RE), Mean Absolute Error (MAE), and Root Mean Square Error (RMSE), along with the coefficient of determination (*R*^2^).

#### 2.4.1 ACF and PACF

Auto-correlation refers to the relationship between a current observation and one from a previous time step (lag) within time series data. An auto-correlation plot, which graphs auto-correlation against lag, visualizes this relationship. The ACF quantifies the linear connection between an observation at time t and another at time t + k. This concept is encapsulated in the time series Equation 9 as follows:


(9) $$ACF\left(Y_{t},Y_{t+k}\right)=\rho \left(Y_{t},Y_{t+k}\right)=\frac{Co\nu \left(Y_{t},Y_{t+k}\right)}{Var\left(Y_{t}\right)}$$

Where *k* represents the lag, it is defined by $Y_{t}$ and $Y_{t+k}$ . Lag *k* auto-correlation describes the correlation between observations that are *k* time periods apart. Conversely, partial auto-correlation measures the correlation between the time series and its lags, but only after removing the influence of observations in between. Essentially, PACF “subtracts” the correlation that earlier lags have already accounted for. This feature is crucial for identifying the appropriate order for the AR model, as illustrated in the Equation 10.


(10) $$PACF\left(Y_{t},Y_{t+k}\right)=Corr\left(Y_{t}-Y_{t}{\;}^{\prime \prime },Y_{t+k}-Y_{t+k}{\;}^{\prime \prime }\right)$$

$Y_{t}{\;}^{\prime \prime }$ represents the estimated value of $Y_{t}$ , derived through linear regression analysis of variables $Y_{t-1}$ , $Y_{t-2},$ …, $Y_{t-k+1}$ . Similarly, $Y_{t+k}{\;}^{\prime \prime }$ signifies the estimated value of $Y_{t+k}$ , calculated using linear regression based on $Y_{t+k-1}$ , $Y_{t+k-2}$ ,…, $Y_{t}$ . $Corr\left(Y_{t}-Y_{t}{\;}^{\prime \prime },Y_{t+k}-Y_{t+k}{\;}^{\prime \prime }\right)$ denotes the correlation coefficient that measures the relationship strength between $Y_{t}-Y_{t}{\;}^{\prime \prime }$ and $Y_{t+k}-Y_{t+k}{\;}^{\prime \prime }$ . $PACF\left(Y_{t},Y_{t+k}\right)$ is defined as the partial auto-correlation coefficient for a lag of k periods.

#### 2.4.2 ADF test

The unit root test stands as the principal technique for assessing the smoothness of time series data. Within the framework of the ADF test, it is essential to conduct a regression analysis to derive the coefficients α , β , and Y , along with the residual error’s variance, followed by a unit root test on the residual term. Absence of a unit root in this term indicates that the time series is smooth, leading to the rejection of the null hypothesis. Conversely, presence of a unit root signifies a non-smooth time series, necessitating the acceptance of the null hypothesis. In practical applications, the ADF test’s *p* value is frequently employed to gauge the time series’ smoothness. A *p* value below the significance threshold (commonly set at 0.05 or 0.01) leads to the rejection of the null hypothesis, indicating that the time series is indeed smooth. The Formula 11 is described as follows:


(11) $$Y_{t}-Y\left(t+k\right)=\alpha Y\left(t+k\right)+\beta_{t}+\gamma +\varepsilon t$$

Where $Y_{t}$ represents the initial observation in the time series, Y(t+k) denotes a subsequent observation, α is the regression coefficient, β refers to the coefficient of the time trend, γ stands as the intercept (the point at which a line crosses the x- or y-axis), and γ signifies the error term (in statistics).

#### 2.4.3 Akaike’s information criterion

To elucidate the connections among variables, it is crucial to assess the generated models for their performance efficacy. This evaluation employs insightful criteria to gauge a model’s ability to delineate a relationship accurately. The primary metric used is the AIC, which evaluates model quality by rewarding models that minimize errors and penalizing those with excessive parameters. AIC is mathematically formulated as follows (Equation 12).


(12) AIC=−2LL+2K

LL represents the log-likelihood function, and K stands for the number of parameters. Within the AIC framework, a lower score signifies the optimal model, characterized by a greater likelihood value. This aids the time series analyst in choosing the most suitable model from a restricted pool of possible models.

#### 2.4.4 LB test

The LB test evaluates the presence of auto-correlation in a time series, examining not only white noise in the residuals but also testing for complete randomness, as implied by the nature of white noise, where residuals are uncorrelated. The test uses the *p* value to assess if the series is independent, random, and exhibits white noise characteristics. A *p* value below 0.05 leads to the rejection of the null hypothesis, suggesting the series is correlated; whereas a *p* value of 0.05 or higher indicates acceptance of the null hypothesis, portraying the series as an uncorrelated white noise sequence. This is represented in Equation (13).


(13) Q=n(n+2)∗∑(r^2)(n−k)

Where *n* represents the sample size, *r* stands for the auto-correlation coefficient of the residual series, and *k* denotes the order of the auto-correlation coefficient. By referring to the critical values of *Q* and the degrees of freedom, one can conduct hypothesis testing to ascertain the presence of auto-correlation in the residual series.

#### 2.4.5 Evaluation metrics

RE, MAE, RMSE, and *R*^2^ are frequently utilized to assess the accuracy of the proposed model, as described by Equations (14–17). The *R*^2^ value varies between 0 and 1, with values closer to 1 indicating a better model fit. Conversely, for RE, a smaller RMSE and MAE suggest a more accurately fitted model.


(14) $$R^{2}=1-\frac{\sum {\left(Y,i-{\hat{Y}}_{i}\right)}^{2}}{\sum {\left(Y,i,-,Y_{i}\right)}^{2}}$$

(15) $$RE=\left|\frac{\left(Y,i-{\hat{Y}}_{i}\right)}{{\hat{Y}}_{i}}\right|*100\%$$

(16) $$RMSE=\sqrt{\frac{1}{n}}\overset{n}{\underset{i=1}{\sum }}{\left({\hat{Y}}_{i}-Y{,}_{i}\right)}^{2}$$

(17) $$MAE=\frac{1}{n}\overset{n}{\underset{i=1}{\sum }}\left|{\hat{Y}}_{i}-Y{,}_{i}\right|$$

Where Y,i denotes the actual value, $\hat{Y}i$ signifies the forecasted value, $Y_{i}$ stands for the average value, and n indicates the sample size.

### 2.5 Statistical methods

**Figure 1:**
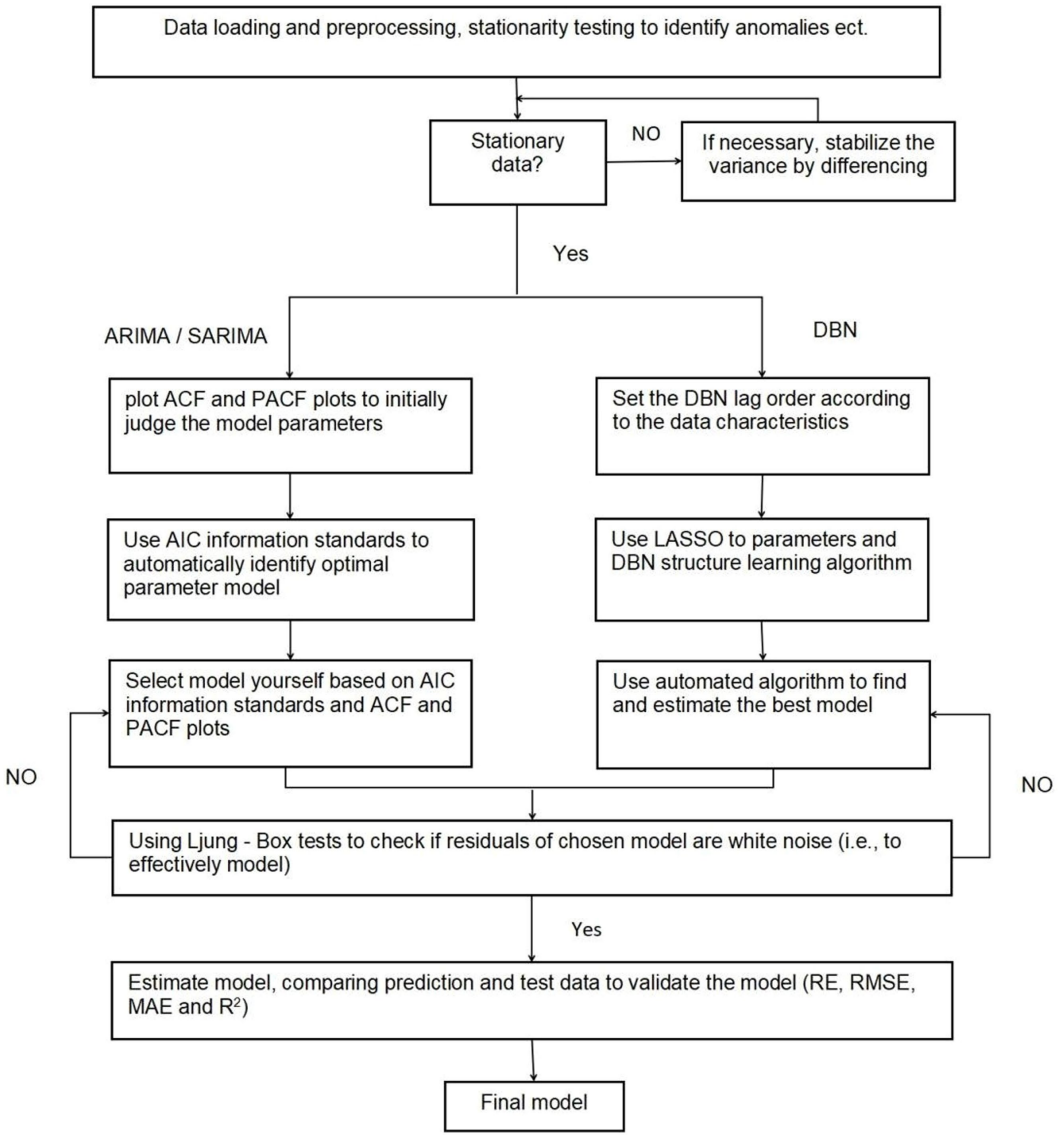
Algorithm demonstrating the approach for constructing ARIMA, SARIMA, and DBN models.

Data analyses were conducted using R version 4.3.1, utilizing the “forecast,” “tseries,” “lars,” and “bnlearn” packages. Hypothesis testing was performed with a significance level of 0.05. For the analysis steps, refer to Figure 1.

#### 2.5.1 ARIMA and SARIMA modeling

1. Employ the ADF unit root test to determine if the maritime emergency time series data is smooth or stationary, differencing any non-smooth information.
2. Generate auto-correlation and partial correlation plots of the maritime emergency time series to initially evaluate appropriate model parameters.
3. Fit possible ARIMA/SARIMA models according to step 2 and select the optimal model. Use the LB test to determine whether the residuals of the optimal model are white noise, and only the model whose residuals are white noise is a valid model.
4. Predictions utilized the optimal model, calculating RE, RMSE, MAE, and R^2^ to compare the models’ predictive efficiency.

#### 2.5.2 DBN modeling

1. Assess the smoothness of the maritime emergency time series data. If non-smooth, apply differencing before model fitting.
2. Determine the lag order of the DBN based on data characteristics. Utilize the LASSO algorithm to learn DBN parameters and structure.
3. The potential DBNs were adjusted as per step 2, and the best model was chosen. Subsequently, the LB test was applied to check if the residuals of the chosen model exhibit white noise characteristics, thereby confirming the model’s validity.
4. Utilize the optimal model for predictions. Calculate the RE, RMSE, MAE, and R^2^ to evaluate model performance. Compare the predictive effectiveness of the models.

## 3 Results

### 3.1 Descriptive analysis

**Figure 2:**
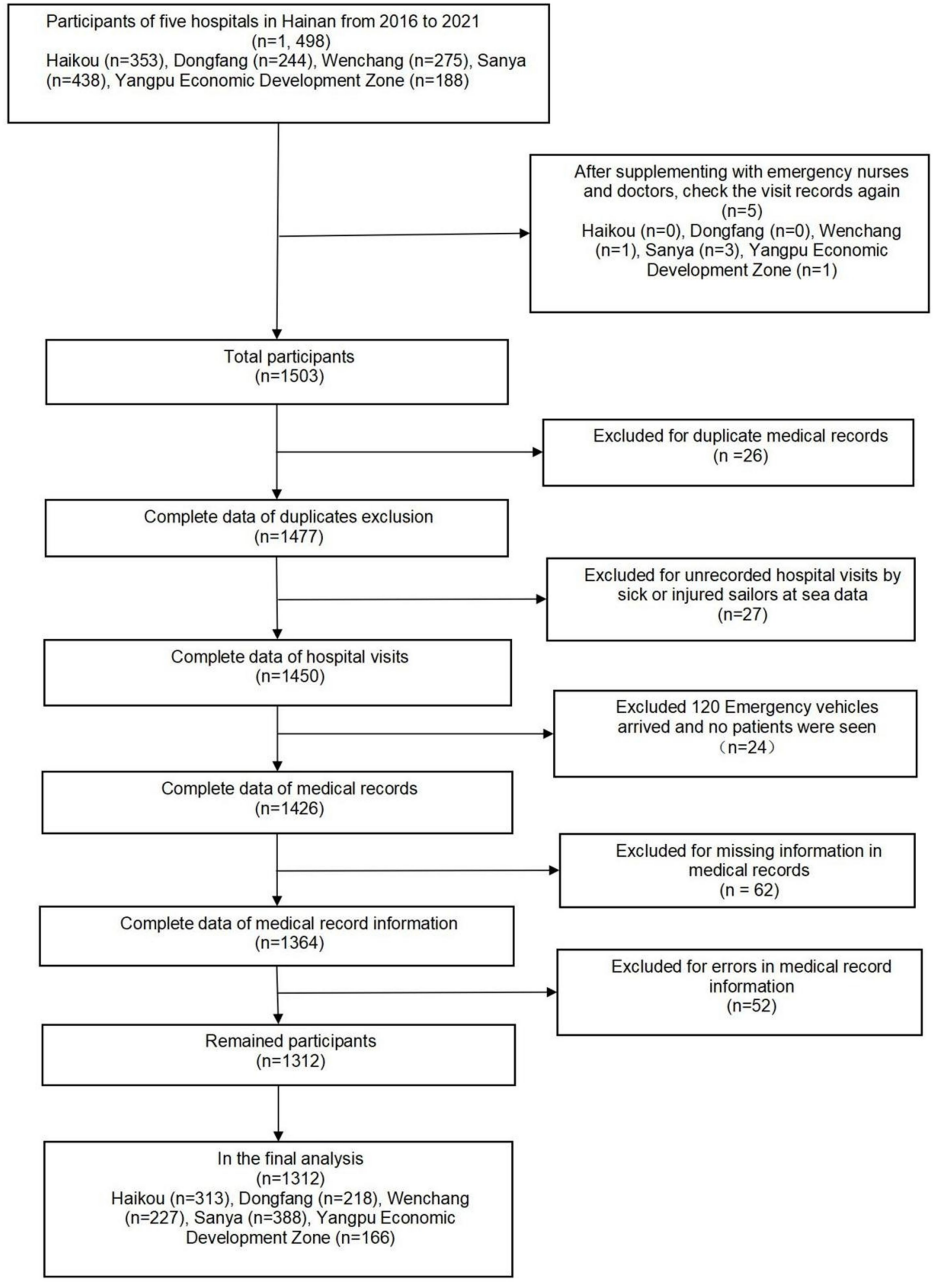
Flowchart of case screening.

From January 2016 to December 2021, five hospitals in Hainan Province documented 1,312 maritime emergencies, with an average of 18.22 incidents per month. The process of case selection is outlined in Figure 2. The 60 time points of maritime first aid from January 2016 to December 2020, as detailed in Table 1, were inputted into *R* and depicted in a trend chart. Figure 4 displays the fluctuations in the number of maritime first aid incidents in Hainan Province during this timeframe. The data were preprocessed to decompose a series of factors influencing changes in maritime first aid in the South China Sea, as depicted in Supplementary Figure 2. Figure 4 and Supplementary Figure 2 present the time series analysis of maritime first responders in Hainan, unveiling a noticeable seasonal pattern. October, November, and December emerge as peak months for rescue and treatment services, while June and July experience the lowest activity, likely attributed to the summer season.

**Figure 3:**
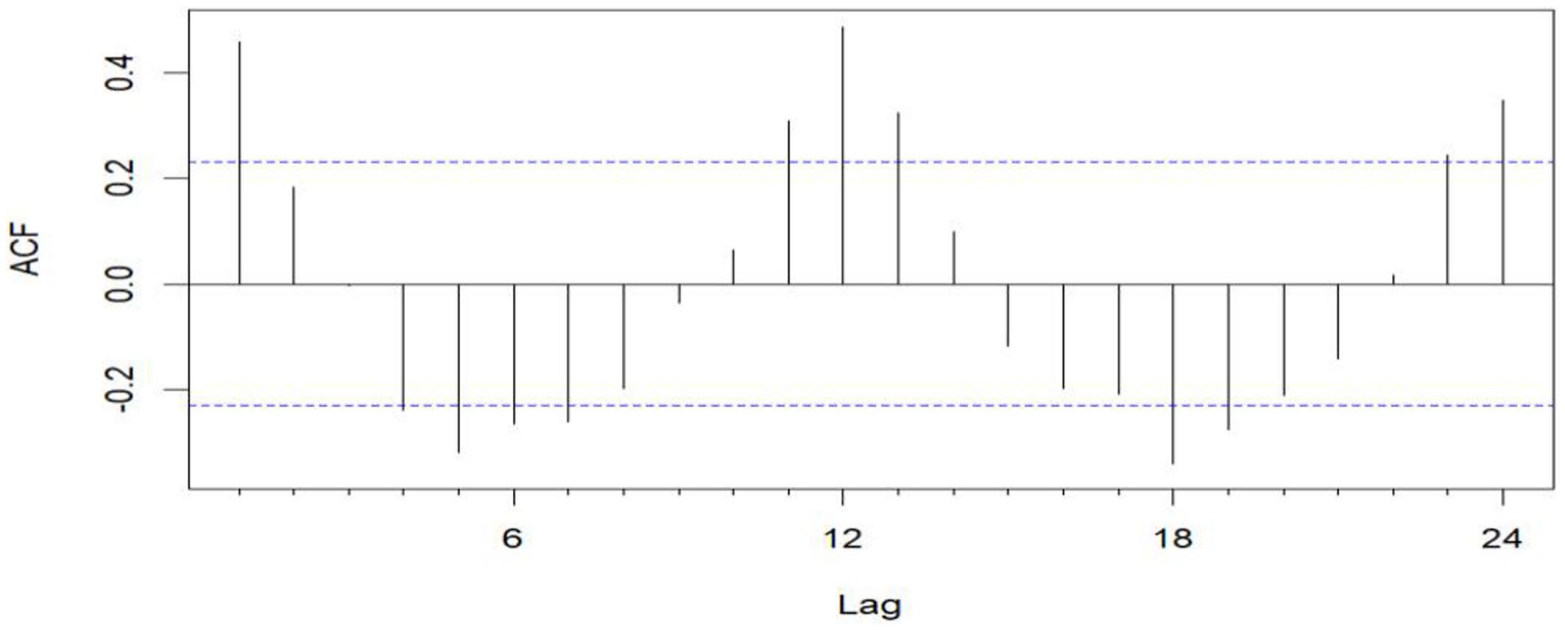
Sequential ACF chart of the number of emergency cases at sea from January 2016 to December 2020.

**Figure 4:**
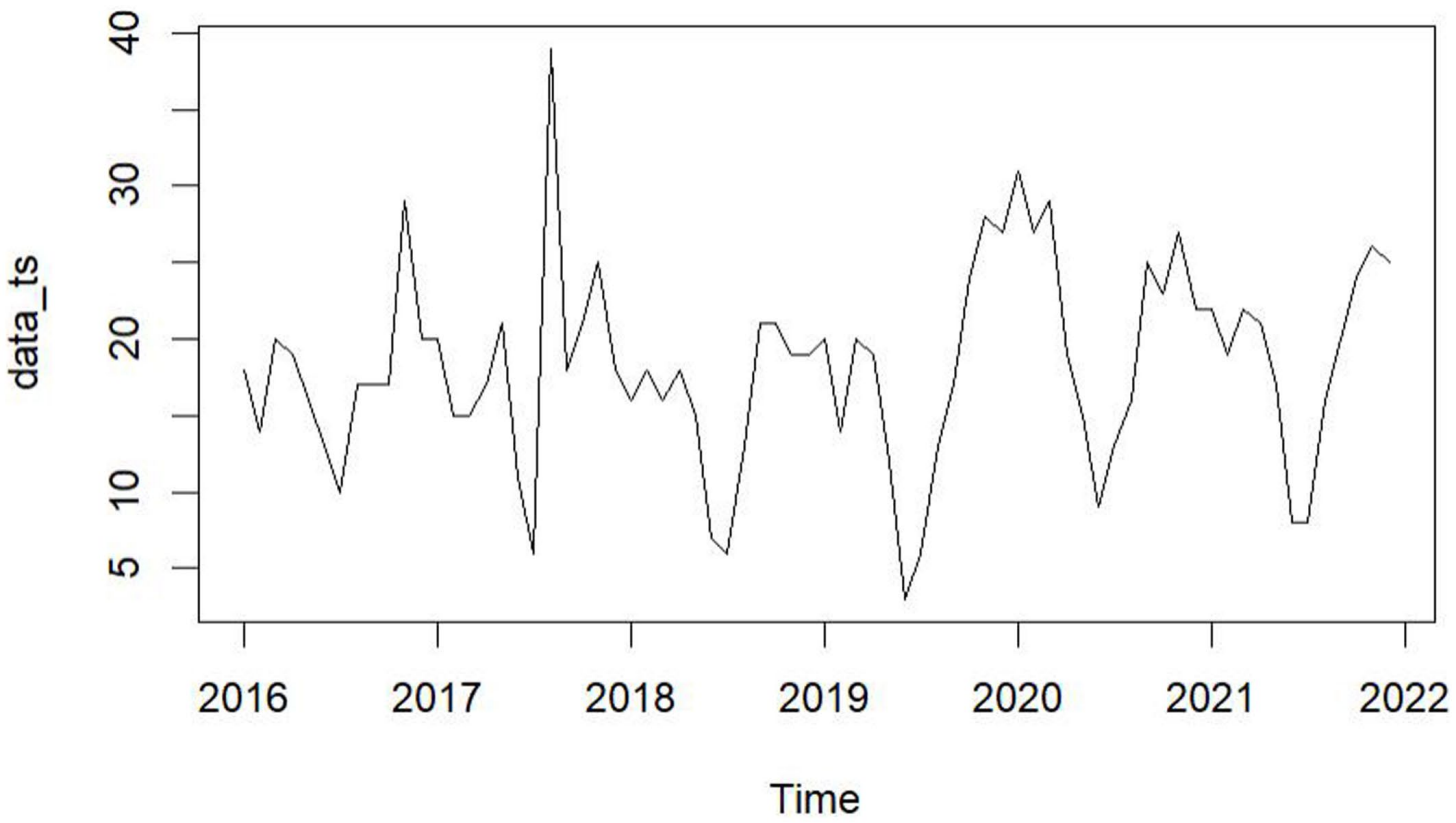
Sequence of maritime EMS attendances from January 2016 to December 2021.

#### 3.1.1 ARIMA modeling

**Figure 5:**
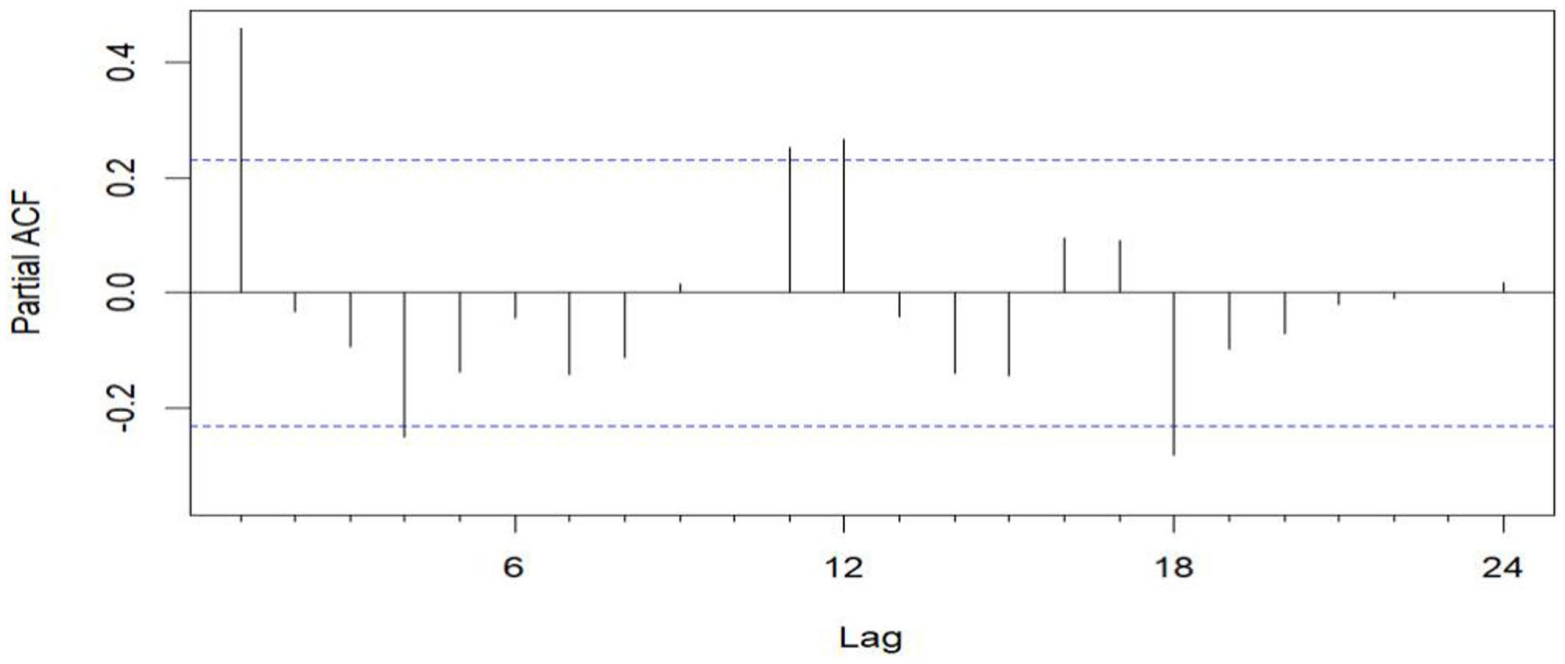
Sequential PACF chart of the number of EMS cases at sea from January 2016 to December 2020.

The unit root test for the count of sea-based first responders from January 2016 to December 2020 yields ADF = 0.01, *p* ≤ 0.01, confirming the series’ stationarity. Figures 3, 5 illustrate the ACF’s sinusoidal trend and the PACF’s initial spike, followed by diminishing lags, suggesting an ARIMA (1, 0, 0) model. However, model selection can be subjective. Objectively, the AIC criterion prefers the ARIMA (1, 0, 0) model, with the lowest AIC at 462.8274 (see Table 2) and an *R*^2^ value of 0.21. The LB test result, *P*_LB text_ = 0.8695, exceeding 0.05, indicates the residuals are white noise, validating the model’s predictive accuracy for the number of sea-based first responders.

**Table 2:** ARIMA model least squares parameter estimation.

Model	AIC
ARIMA (2,0,2)	469.6173
ARIMA (0,0,0)	477.6239
ARIMA (1,0,0)	462.8274
ARIMA (0,0,1)	465.1882
ARIMA (0,0,0)	632.9414
ARIMA (2,0,0)	465.0171
ARIMA (1,0,1)	465.0313
ARIMA (2,0,1)	Inf
ARIMA (1,0,0)	483.6405

#### 3.1.2 SARIMA modeling

**Figure 6:**
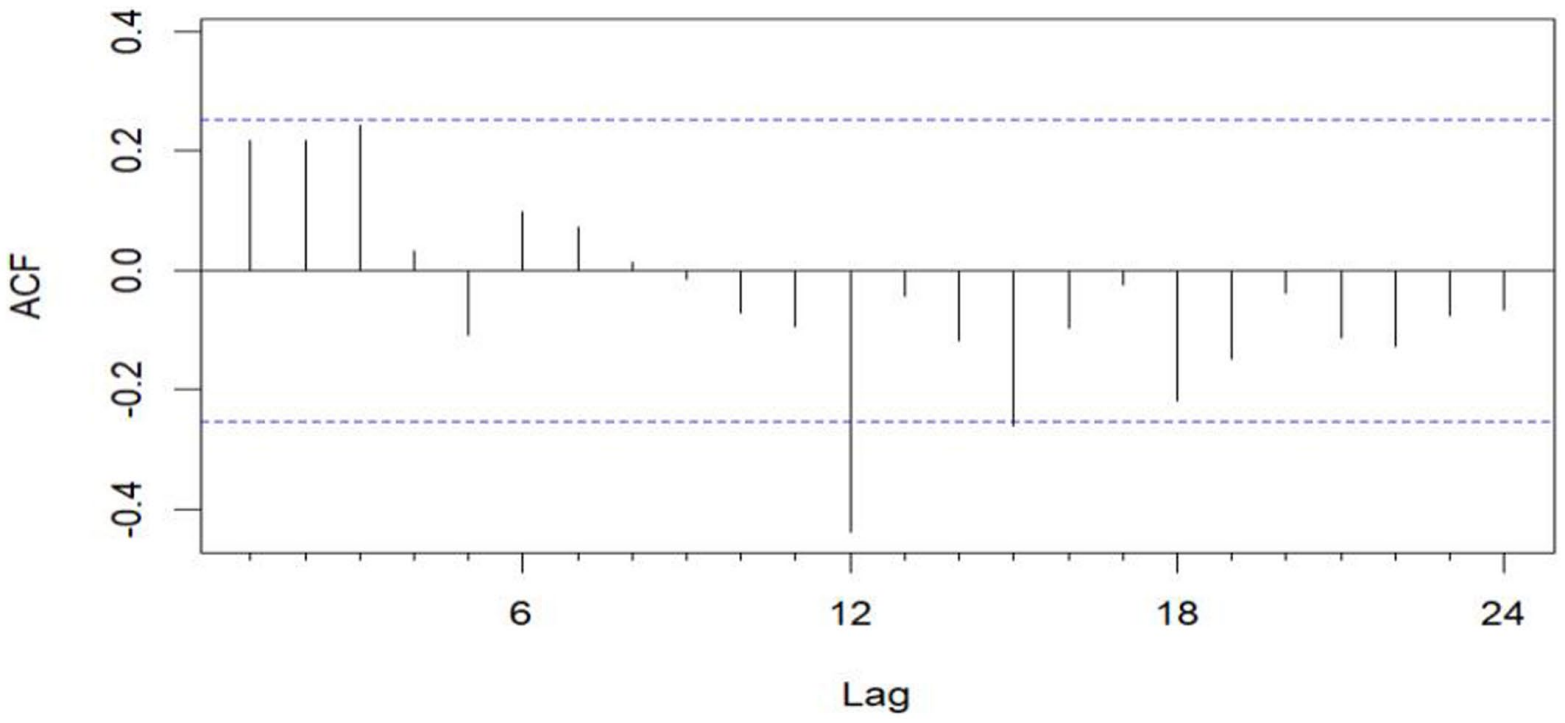
ACF plot after differential.

**Figure 7:**
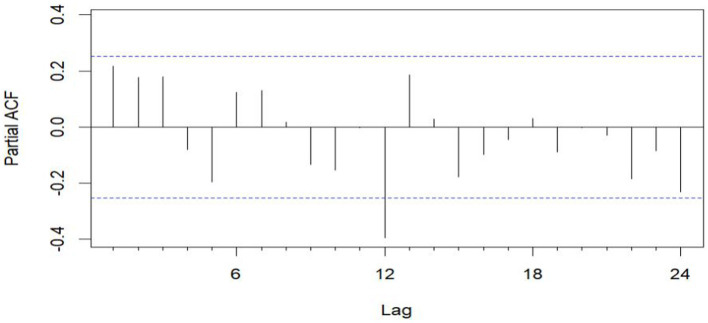
PACF plot after differential.

**Figure 8:**
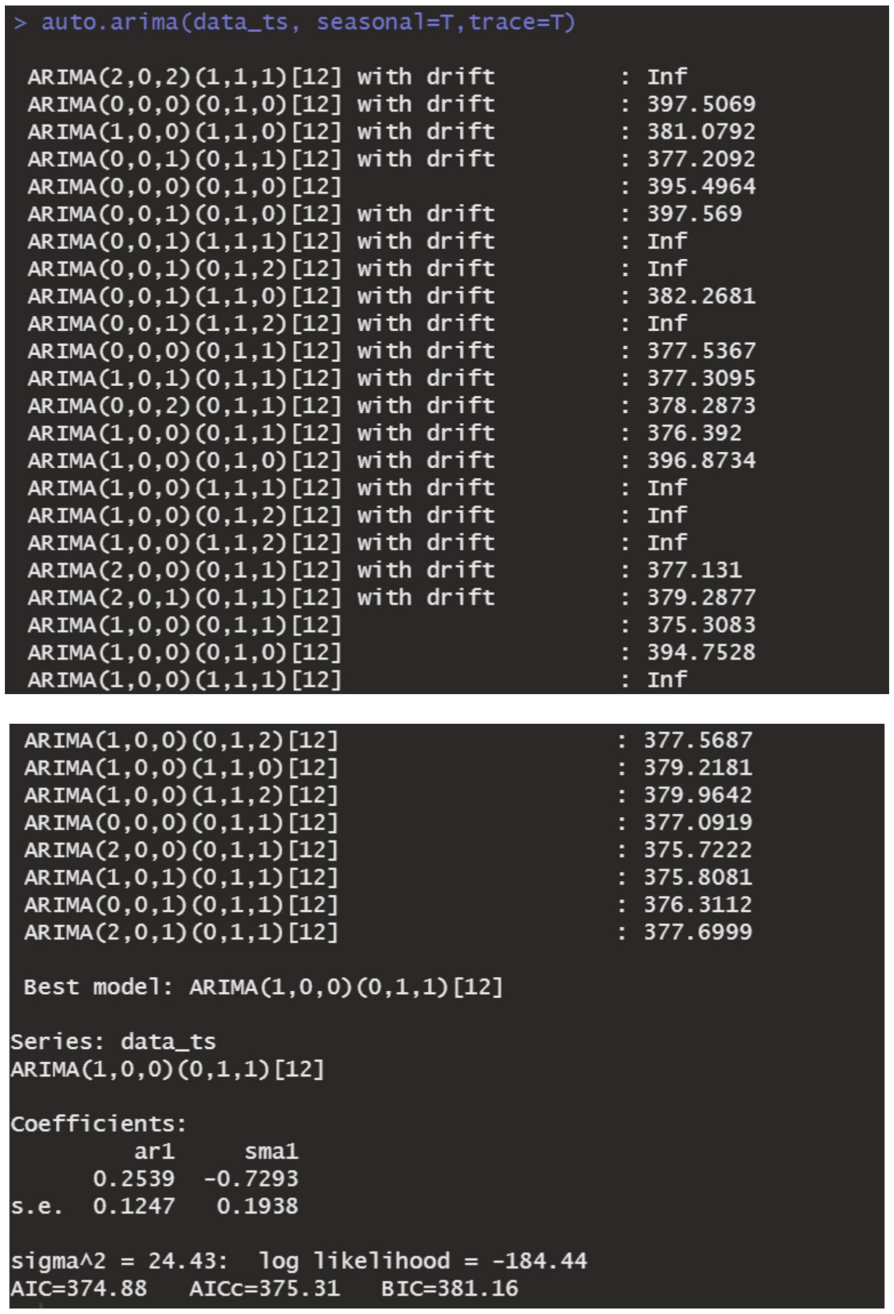
Results of AIC parameter estimation.

The data remains consistent, yet the time series graph reveals a distinct seasonal trend. As a result, we implemented first-order seasonal differencing. The SARIMA model is expressed as: ARIMA (*p*, *d*, *q*) (*P*, *D*, *Q*)_[*s*]_, where *s* denotes the seasonal cycle, (*p*, *d*, *q*) represents the non-seasonal component of the model, and (*P*, *D*, *Q*)_[*s*]_ denotes the seasonal part of the model. The seasonal cycle is 12 months per year (evident from the spikes at lags 12 in the ACF plot after differentiating the SARIMA model in Figure 6), hence *s* = 12, *D* = 1. By examining the auto-correlation and partial correlation of the maritime EMS trips time series (Figures 6, 7) and using the AIC for model selection, we identified SARIMA(1,0,0)(0,1,1)_12_ as the optimal model. The AIC’s parameter estimation results are depicted in Figure 8. This model boasts an *R*^2^ value of 0.54, and the LB test, with a *p* value of 0.6729—above the 0.050 threshold—verifies that the residuals are white noise, underscoring the model’s precision and reliability in predicting the number of maritime first-aiders.

#### 3.1.3 DBN modeling

**Figure 9:**
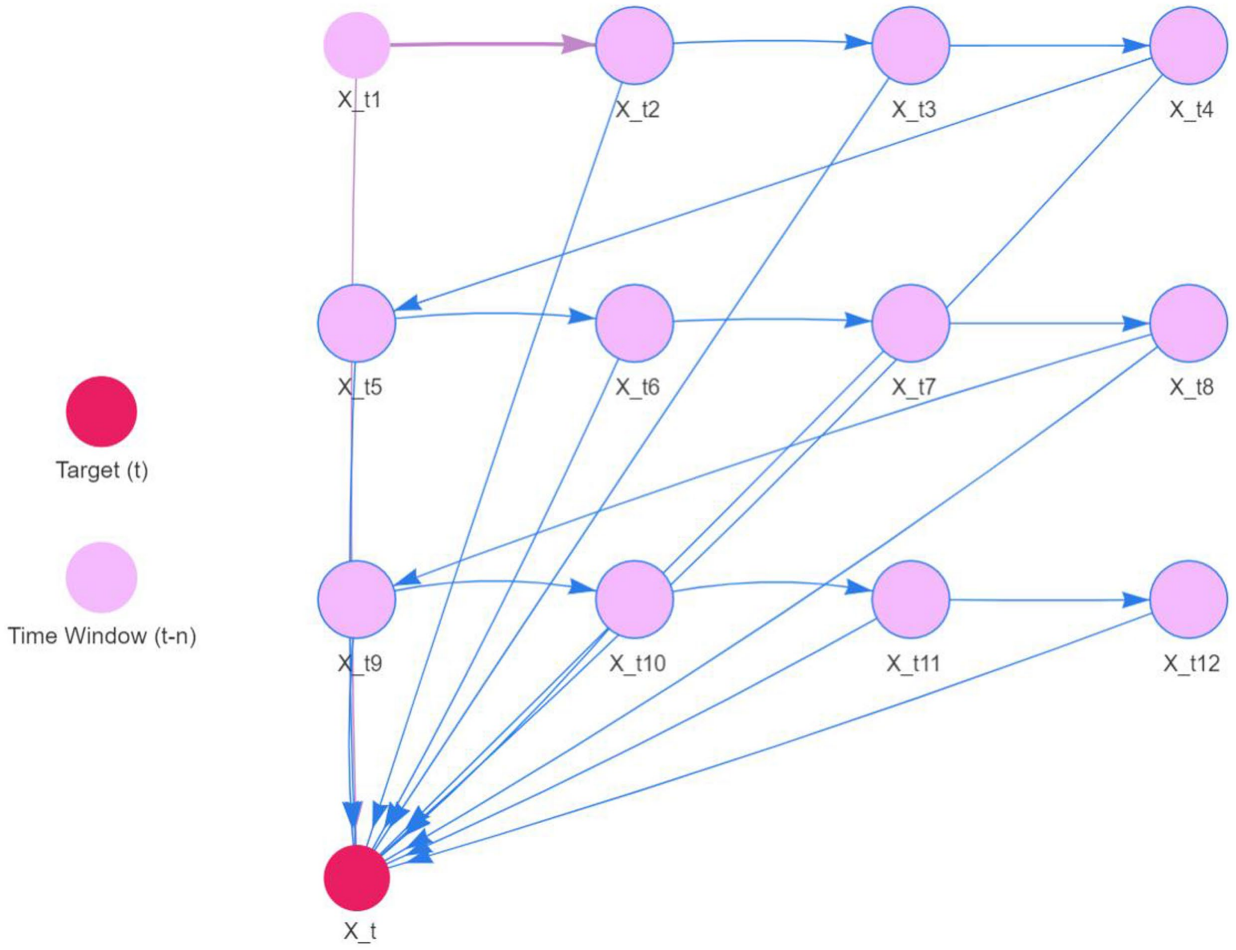
DBN model of first aid trips at sea.

The original series is renowned for its fluidity. Consequently, the DBN model was precisely tailored with a maximum lag of 12 to accommodate seasonality. We utilized 12 sequences of lagged morbidity numbers, ranging from 1 to 12, as input. The LASSO algorithm facilitated parameter estimation, with outcomes detailed in Table 3. The nonzero coefficients of the 12 lagged morbidity numbers suggest a significant correlation with the current period’s maritime first-aiders count. This relationship is mirrored in the DBN’s structure, illustrated in Figure 9. The DBN’s *R*^2^ stood at 0.44. Moreover, the LB test yielded a *p* value of 0.708, surpassing the 0.050 threshold, with the residuals classified as white noise, affirming the model’s predictive accuracy for the number of sea first responders.

**Table 3:** Results of DBN modeling parameter estimation.

Lag	Coefficient
X_t1	0.31678192
X_t2	0.19788793
X_t3	−0.05581918
X_t4	0.06195344
X_t5	−0.10013309
X_t6	−0.04453516
X_t7	0.02913787
X_t8	−0.08300831
X_t9	−0.07589329
X_t10	0.04222040
X_t11	0.04748903
X_t12	0.28105241

#### 3.1.4 Comparison of three model fitting and prediction effects

**Figure 10:**
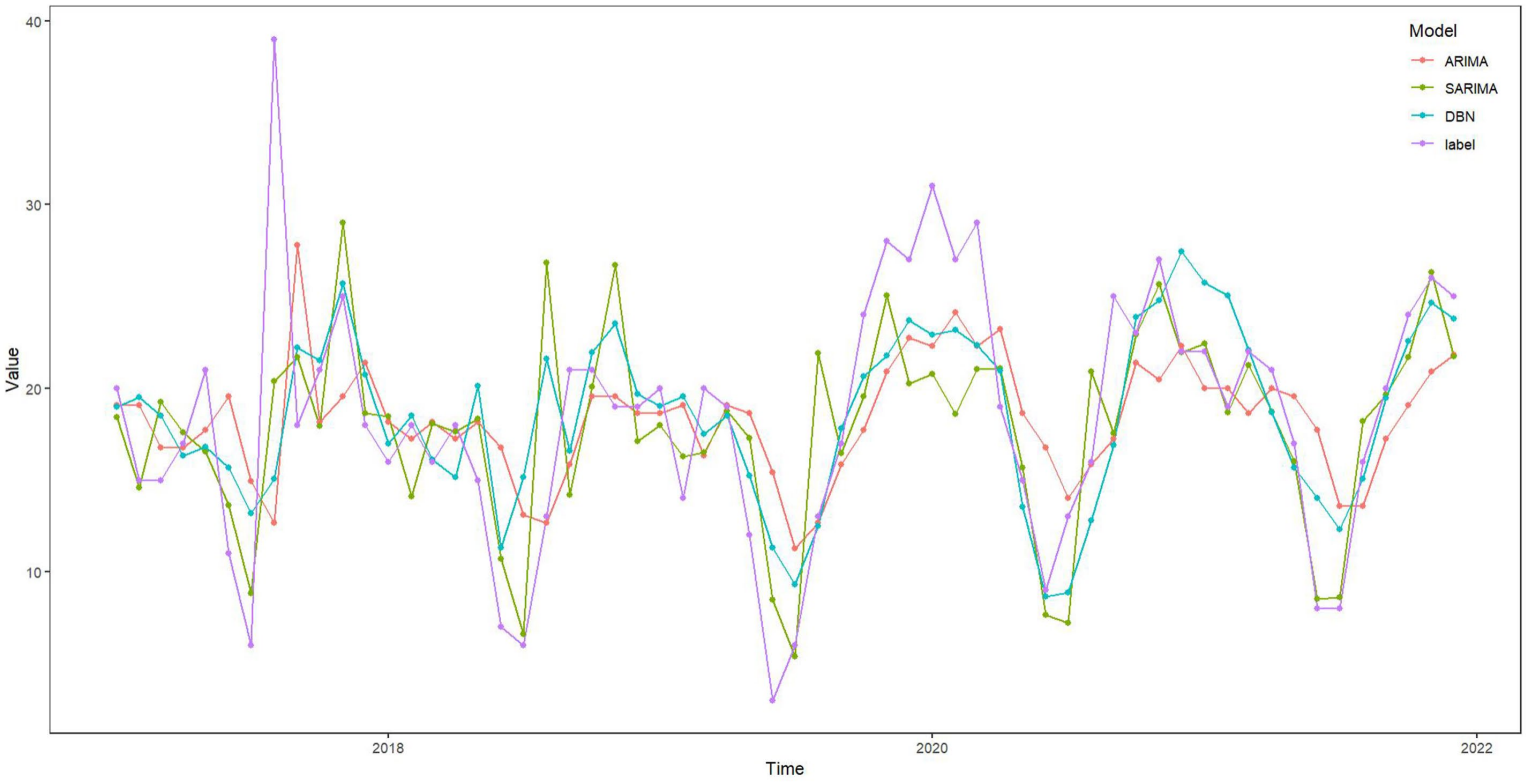
Fitting and prediction plots of ARIMA, SARIMA, and DBN models.

The ARIMA, SARIMA, and DBN models were utilized for forecasting. Table 4 showcases the forecasted outcomes from each model, with SARIMA’s forecasts more accurately mirroring actual rescues than those of ARIMA and DBN. Following this, the RMSE and MAE for each model were computed and are detailed in Table 5. The *R*^2^ scores for ARIMA, SARIMA, and DBN range from 0 to 1, with SARIMA’s score surpassing 0.5, signifying a robust model fit. Hence, SARIMA demonstrates superior performance over the ARIMA and DBN models. Specifically, SARIMA’s RMSE and MAE saw reductions of 22.96 and 31.96%, respectively, in comparison to ARIMA. Additionally, compared to ARIMA, SARIMA’s RMSE and MAE fell by 18.72 and 27.01%, respectively, whereas DBN’s RMSE and MAE saw smaller declines of 5.22 and 6.78%, respectively. Figure 10 illustrates the fitting and prediction plots, indicating that SARIMA’s fitting and forecast trajectories more closely match the actual data.

**Table 4:** ARIMA, SARIMA, and DBN projections of the number of patients in maritime emergencies in Hainan from January 2021 to December 2021.

Month	Actual	ARIMA	RE	SARIMA	RE	DBN	RE
Jan.	22	20	9.09%	22	0.00%	26	18.18%
Feb.	19	20	5.26%	19	0.00%	25	31.58%
Mar.	22	19	13.64%	21	4.55%	22	0.00%
Apr.	21	20	4.76%	19	9.52%	19	9.52%
May	17	20	17.65%	16	5.88%	16	5.88%
Jun.	8	28	250.00%	9	12.50%	14	75.00%
Jul.	8	14	75.00%	9	12.50%	12	50.00%
Aug.	16	14	12.50%	18	12.50%	15	6.25%
Sep.	20	17	15.00%	20	0.00%	19	5.00%
Oct.	24	19	20.83%	22	8.33%	23	4.17%
Nov.	26	21	19.23%	26	0.00%	25	3.85%
Dec.	25	22	12.00%	22	12.00%	24	4.00%

**Table 5:** Evaluation metrics for ARIMA, SARIMA, and DBN model fitting and prediction accuracy.

Model	RMSE	MAE	*R* ^2^
ARIMA	5.75	4.13	0.21
SARIMA	4.43	2.81	0.54
DBN	5.45	3.85	0.44
Percentage decrease in SARIMA over ARIMA	22.96%	31.96%	−157.14%
Percentage decrease in SARIMA over DBN	18.72%	27.01%	−22.72%
Percentage decrease in DBN over ARIMA	5.22%	6.78%	−109.52%

## 4 Discussion

In our study, we constructed several forecasting models, including ARIMA, SARIMA, and DBN, to predict the monthly influx of maritime patients treated by hospitals across Hainan Province. By analyzing the treatment data of patients from the South China Sea region, we evaluated and compared the predictive accuracy of these models. Our results reveal that the SARIMA (1, 0, 0) (0, 1, 1)_12_ model outperforms others in forecasting accuracy.

In our study, we analyzed data on maritime emergency patients rescued in the South China Sea, specifically within the Hainan region, from 2016 to 2021. This data, totaling 1,312 rescues, aligns closely with figures reported by the Hainan Maritime Bureau, encompassing police responses and search and rescue operations (31). Our dataset comprises notable events that influenced these figures, including the onset of the COVID-19 epidemic in 2019, Typhoon Pigeon in August 2017, and Super Typhoon Rey in late December 2021. These occurrences resulted in a surge in rescue operations, consistent with our collected data. This correlation, particularly in light of the unpredictable nature of such disasters, emphasizes the accuracy of our predictive model and the objective, realistic selection of our data.

Aside from occasional spikes in Maritime EMS volume resulting from major disasters, there are noticeable seasonal fluctuations and trends. Peak periods for rescue and treatment operations typically occur during the winter months of October, November, and December, while quieter months are observed in June and July. Similar seasonal patterns have been identified in other studies (32, 33). However, both the DBN model and the time series model effectively capture these seasonal fluctuations and trends in rescue numbers, serving as dynamic tools for forecasting data with periodic characteristics.

Prior research suggests that at least 3 years of historical data are necessary to meet sample size requirements (25), as insufficient data may diminish auto-correlation and hinder the extraction of periodic features, ultimately impacting prediction accuracy (34). This study relies on authentic, objective, and reliable medical records from Hainan hospitals specializing in maritime emergency care. The dataset spans 5 years and 60 months from 2016 to 2020, constituting a complete cycle and meeting the prerequisites for modeling. To the best of our knowledge, this is the first study to apply a DBN model and time series analysis to predict maritime EMS dispatches in China.

To enhance the management of first aid responders at sea, it is crucial to improve sea rescue prevention and control strategies, necessitating cooperation from all stakeholders. Accurately predicting emergency incidents at sea is vital for effective sea rescue operations. This study aims to provide predictive analytics for managing maritime first aid in the South China Sea. After extensive analysis, we identified the ARIMA (1, 0, 0) and SARIMA (1, 0, 0) (0, 1, 1)_12_ models as optimal. By developing the DBN model and refining it through iterative debugging based on data characteristics, we established a DBN model with a maximum lag of 12 orders, starting with a non-zero lag order coefficient. Comparing the ARIMA (1, 0, 0) and SARIMA (1, 0, 0) (0, 1, 1)_12_ models’ fitting capabilities with the DBN model, using predicted values, RE, RMSE, MAE, and *R*^2^, revealed that the SARIMA (1, 0, 0) (0, 1, 1)_12_ model offers more precise predictions, lower RE, and better RMSE, MAE, and *R*^2^ scores, outperforming the DBN model. The ARIMA (1,0,0) model showed lesser fitting capabilities. Therefore, the SARIMA (1,0,0)(0,1,1)_12_ model is more effective for forecasting future maritime EMS operations, leading to more accurate EMS trend forecasts.

Numerous studies indicate that DBN methods deliver strong performance in predictive analytics (25, 35), yet their application in the medical sector, including areas like infectious diseases, outpatient volume, and emergency care, remains limited. DBN, a graphical model, effectively represents fitting results through network graphs. DBN estimation employs various machine learning algorithms, notably the LASSO algorithm in this research, along with James-Stein shrinkage estimation and first-order conditional dependence approximation (30, 36, 37). Despite this, the SARIMA method is also recognized for its robust fitting and predictive capabilities (34, 38, 39). Given our data’s specific characteristics, we found SARIMA to surpass both the DBN and ARIMA methods in performance. The SARIMA model excels at capturing and predicting seasonal components, making it effective for handling abnormal and fluctuating data with periodicity (40). This capability is particularly valuable for maritime emergency rescue data, which inherently follows a cyclical pattern. In contrast to the high computational demands and complexity of DBN (41), SARIMA offers high prediction accuracy at lower computational costs, making it well-suited for various real-time and near-real-time applications. While ARIMA struggles with seasonal data (15), SARIMA stands out for its ability to make precise predictions in complex datasets without the need for intricate assumptions or extensive prior knowledge. As illustrated in Figure 10, the comparison of the three models demonstrates how SARIMA (1,0,0)(0,1,1)_12_ adeptly fits historical data, offering a reliable forecast for the number of EMS sea trips.

Forecasting maritime emergency visits is crucial for managing healthcare in coastal areas. Emergency department visits serve as a vital measure of workload and the quality of care provided. Overcrowding occurs when the demand from patients surpasses the available resources during peak times (33). Thus, it is imperative to efficiently allocate medical staff. Precise predictions of maritime emergencies are essential for distributing hospital emergency resources effectively, ensuring the quality and safety of medical services, and optimizing the use of human, financial, and material resources for better economic and social outcomes. Failure to do so may result in the squandering of resources. Based on the findings, actionable steps include: (1) Boosting medical staff reserves in coastal hospitals from September to November, organizing medical staff more logically, and dynamically managing them according to the off-peak season characteristics to leverage their flexibility, adaptability, and synergy. (2) Adopting a flexible scheduling system to reduce work pressure and mental stress on team members, thus safeguarding their well-being and mitigating adverse effects. (3) Enhancing medical resource allocation by opening a fast-track process for resource approval during high-demand periods and allowing resource managers to review and augment resources in slower periods. This not only aids in the training and development of medical staff but also ensures that rescue organizations are adequately prepared for peak season challenges, thereby advancing the precise and sophisticated management of medical resources.

This study introduced the time series model and DBN model into maritime emergency medical rescue research, expanding the scope of the time series model. Through a systematic evaluation of ARIMA, SARIMA, and DBN models, it compares the effectiveness of different time series forecasting models for the number of first responders at sea, demonstrating their application in real-world scenarios. This provides practical cases for maritime administration officials and medical rescue personnel, enhancing the research’s practical application value and laying a foundation for future development in emergency medical management for maritime rescue work. In the future, the prediction model constructed in this study can be extended to other coastal cities. Managers can combine SARIMA with machine learning models (such as Long Short-Term Memory) based on local data, leveraging the strengths of different models to improve prediction accuracy. Moreover, with improved computing power, future research can develop real-time prediction systems, using SARIMA and other efficient models for real-time data analysis and prediction. This will enhance marine monitoring and emergency response efficiency, providing new perspectives and methods for research combining emergency medicine and public health.

The study’s policy recommendations are as follows: (1) Establish a prediction and early warning system. Medical and maritime managers should create a system based on the study’s model. Each unit and institution can regularly upload emergency and environmental data, build a data-sharing platform, and improve data comprehensiveness and timeliness. (2) Enhance the dispatch of emergency rescue resources at sea. Based on predicted personnel needs, dynamically and scientifically allocate rescue vessels, medical supplies, and personnel to ensure sufficient and equitable distribution of resources and reduce response time. (3) Advance intelligent emergency management. Develop a dispatching system that integrates ARIMA, SARIMA, and DBN models into the emergency platform to achieve intelligent dispatching and optimized resource management. Conduct regular emergency drills to validate the prediction model’s accuracy and the dispatching strategy’s feasibility, continuously refine the emergency plan, and enhance overall emergency preparedness.

However, this research encounters two primary constraints. Firstly, it did not encompass the entire Hainan region, resulting in unavoidable data omissions. Secondly, the study compiled only 1,312 medical records, a quantity considerably lower than that in similar studies. This reduced data pool likely impacted the predictive precision of the DBN model compared to the SARIMA model. To achieve more reliable long-term predictions, expanding data collection, collaborating with various centers, and closely examining the time distribution patterns of maritime emergency medical services are essential. Such initiatives will foster more efficient and scientifically accurate strategies for allocating medical staff. Another limitation is the study’s lack of access to detailed environmental and meteorological data, which could have illuminated fluctuations in the need for first responders at sea. Enhancing the proposed model with this additional data could lead to improvements.

## 5 Conclusion

This study marks the first predictive analysis of maritime emergency medical personnel incidents in the South China Sea, specifically within China’s Hainan region, covering the period from 2016 to 2021. It was found that the SARIMA, DBN, and ARIMA models are all effective in forecasting the need for emergency medical treatment at sea. Among these, the SARIMA method stood out for its superior accuracy over the DBN and ARIMA approaches. By applying the SARIMA (1, 0, 0) (0, 1, 1)_12_ model to forecast the number of first aid responders at sea, this research provides valuable scientific insights for policymakers in management. It supports dynamic training of personnel, planning for human resource allocation, and optimizing resource use.

## Data availability statement

The raw data supporting the conclusions of this article will be made available by the authors, without undue reservation.

## Ethics statement

The studies involving humans were approved by Hainan Medical University Ethics Committee (NO.: HYLL-2022-018). The studies were conducted in accordance with the local legislation and institutional requirements. Written informed consent for participation in this study was provided by the participants’ legal guardians/next of kin. Written informed consent was obtained from the individual(s), and minor(s)’ legal guardian/next of kin, for the publication of any potentially identifiable images or data included in this article.

## Author contributions

PY: Conceptualization, Data curation, Formal analysis, Investigation, Writing – original draft. PC: Conceptualization, Data curation, Formal analysis, Investigation, Writing – original draft. NZ: Investigation, Writing – review & editing. DL: Investigation, Writing – original draft. BX: Validation, Writing – review & editing. HZ: Funding acquisition, Project administration, Supervision, Validation, Writing – review & editing.

## Glossary

COVID-19: Corona Virus Disease 2019
U.K.: United Kingdom
EMS: Emergency medical services
ARIMA: Auto-regressive Integrated Moving Average
SARIMA: Seasonal Auto-regressive Integrated Moving Average
DBNs: Dynamic Bayesian networks
LASSO: Least Absolute Shrinkage and Selection Operator
OLS: Ordinary least square
BNs: Bayesian networks
DAG: Directed acyclic graph
ACF: Auto-correlation function
PACF: Partial auto-correlation function
ADF test: Augmented Dickey-Fuller Test
AIC: Akaike’s information criterion
RE: Relative error
MAE: Mean absolute error
RMSE: Root mean square error
*R* ^2^: The coefficient of determination
MA: Moving Average
AR: Auto-Regressive
LB test: Ljung-Box Test
