# Supplementary figures and images for: Statistical machine learning models for prediction of China’s maritime emergency patients in dynamic: ARIMA model, SARIMA model, and dynamic Bayesian network model
Source: Front Public Health. 2024 Jun 27;12:1401161. doi: 10.3389/fpubh.2024.1401161 (PMC11252837; doi:10.3389/fpubh.2024.1401161)

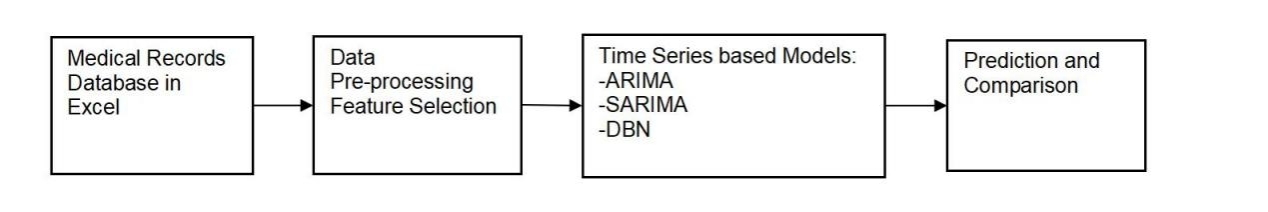

Supplement: Supplementary file 1 [file Image_1.JPEG]

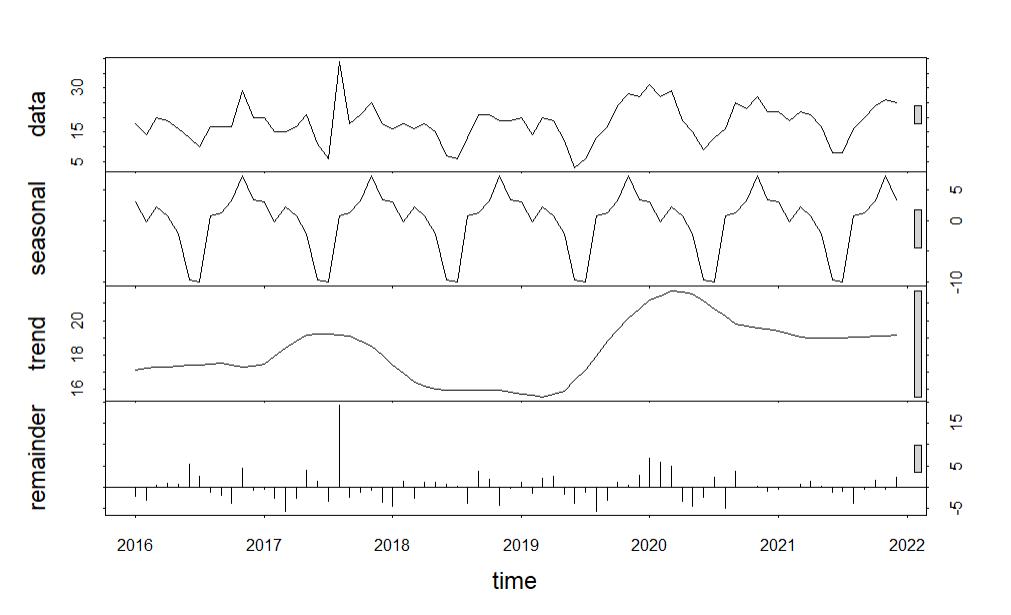

Supplement: Supplementary file 2 [file Image_2.JPEG]
